# Acute median nerve palsy due to hemorrhaged schwannoma: case report

**DOI:** 10.1186/1749-7221-2-19

**Published:** 2007-09-24

**Authors:** Mehmet Dumlu Aydin, Dilcan Kotan, Muzaffer Keles

**Affiliations:** 1 Department of Neurosurgery, Medical Faculty, Ataturk University, Erzurum, Turkey; 2 Neurology Clinic of Batman State Hospital, Batman, Turkey; 3 Department of Pathology, Medical Faculty, Ataturk University, Erzurum, Turkey

## Abstract

Schwannomas are common, benign nerve tumors originating from the sheath of peripheral nerves. In this article, a 54 year old woman suffered from sudden onset motor and sensory deficit at her first radial three fingers on her right hand. Radiological investigations were normal. Electromyography diagnosed a median nerve entrapment neuropathy and urgent surgery was performed. Interestingly, a hemorrhaged mass was detected in the median nevre at the proximal end of the carpal ligament and was resected totally. Histopathological diagnosis was Schwannoma. The patient maintained a healthy status for five years.

## Background

Although peripheral nerve tumors are rare, the median nerve (MN) is one of the most affected peripheral nerves [1]. Schwannomas arising from Schwann cells are usually benign tumors and comprise 0.8% to 2% of all hand tumors [2]. The tumor is usually seen as a painless, asymptomatic mass. Pain, paresthesias and motor weakness may occur when the tumor reaches sufficient size. They are easily separated from surrounding tissues [3]. Lipoma, lipofibroma, hamartoma and intraneuronal hemangioma should be considered in differential diagnosis [4]. Electromyography (EMG) [5], computed tomography (CT), magnetic resonance imaging (MRI) and ultrasonography are very useful in diagnosis [6,7]. Surgical therapy results in excellent results in 90% of patients [1].

## Case Presentation

**Figure 1:**
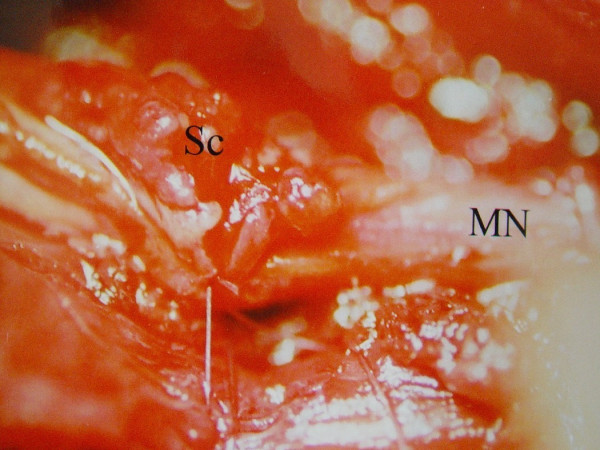
Median nerve (MN) and Schwannoma mass (Sc) are seen intraoperatively.

**Figure 2:**
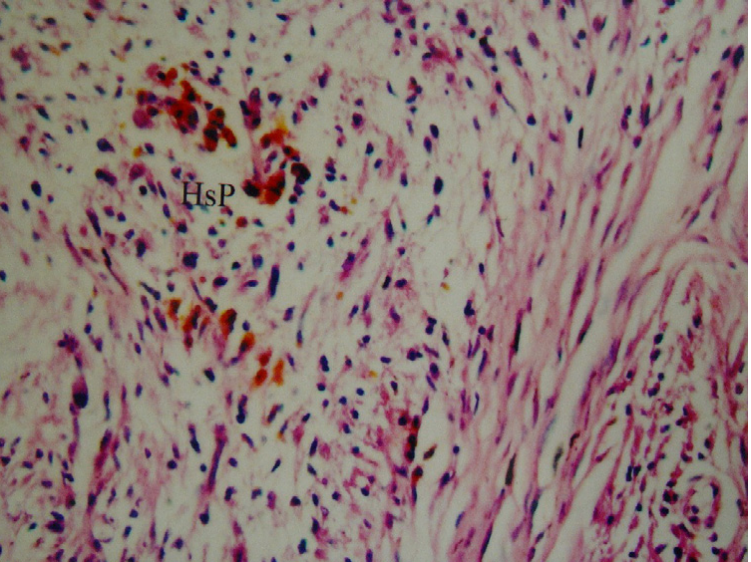
Hypercellular-hypocellular regions, hyalinised blood vessels and hemosiderin pigment (HsP) collections are observed (LM, H&E, ×100).

A 54-year old woman was admitted with a history of abrupt weakness and sensory loss at her radial three fingers on her right hand. She had suffered from pain, aching, burning, tingling, numbness, weakness and clumsiness in the first fingers of her right hand for two months. In neurological examination, sensory loss and flexion paralysis were detected in her radial three fingers. Tinel's sign and Phalen's wrist flexion test were positive. EMG indicated mild median nerve compression at the carpal tunnel with a 4.10 ms distal motor latency and 33.2 m/s sensory nerve conduction velocity of the index finger. MRI did not show a lesion at the course of MN. Carpal tunnel syndrome was considered, and urgent operation was planned. The patient underwent standard carpal tunnel exploration. After release of the transverse carpal ligament, the median nerve was explored. Interestingly, a pulsatile and fusiform bulging was observed on the MN just proximal to the carpal ligament. When the MN sheath was incised along the bulging segment, black cherry juice like fluid leaked spontaneously and a reddish tumoral mass, 2 × 3 mm in diameter, was observed and resected completely without neural lesioning (Fig. 1). Histopathological analysis was Schwannoma (Fig. 2). The patient healed completely three months after surgery.

## Discussion

Tumors of the peripheral nerves are rare [8,9]. Schwannomas arise sporadically and also occur with some forms of neurofibromatosis [8]. Schwannomas are benign, slowly growing, encapsulated neoplasms and are easily separated from the surrounding tissues. Some forms may be localised within nerve trunk or bundles of neurofibrils spreading over the surface of the tumor. Schwannomas can compress the motor and sensorial branches of the MN and may cause aching, burning, tingling, numbness, weakness and clumsiness in the radial half of the hand and radial three digits [10]. They may be easily resected nearly in all cases without causing any complication [3,8].

Schwannomas may be benign or malignant [4]. Histologically, they are composed of two types of cells: The Antony A, which are dense spindle cells, and the Antony B, which are loosely arranged cells [8]. The MN may show hemorrhagic necrosis in some malignant forms of Schwannoma [11]. However, the cause of acute MN palsy in the present case was bleeding of a benign Schwannoma.

In the differential diagnosis, lipoma, lipofibroma, hamartoma and intraneuronal hemangioma must be considered [4]. EMG studies may reveal prolonged sensory latency and diminished or absent sensory evoked potentials [5]. CT and MRI also give useful information regarding tumor extent, anatomical location, tumor size, relationship of peripheral nerve and for appropriate planning of surgical therapy and preoperative diagnosis. Schwannoma is a slightly hypodense, solid tumor with no vascular contrast enhancement on CT. T1-W MRI shows intermediate signals, and T2-W imaging shows high signal intensity with some heterogenity [6,12]. Although CT and MRI can provide useful information about morphological data on the MN tumors, they cannot provide dynamic information. Conversely, ultrasonography gives detailed informative images of MN during static and dynamic positions such as active and passive flexion and extension maneuvers, showing the nerve in relation to the surrounding musculotendinous structures [7].

Surgical excision is the most effective method of therapy, and total recovery is about 90%, though Plexiform neural tumors may exhibit recurrence and malignant transformation in some cases [1,13]. Paresthesia is the most frequent postoperative complication in these patients [1]. Nerve grafting may also be required in some malignant forms of these tumors [11].

## Conclusion

In the presented case, intratumoral hemorrhage was responsible for the acute MN palsy. In carpal tunnel syndrome cases, tumoral lesions should be considered in differential diagnosis. To our knowledge, acute median nerve palsy due to intratumoral Schwannoma hemorrhage has not previously been reported in the literature. This should be added to the list of differential diagnoses of acute MN palsy.

## Competing interests

The author(s) declare that they have no competing interests.

## Authors' contributions

MDA performed surgery, played role in clinical evaluationn and treatment protocol. DK conducted electromyograhy and interpreted results. MK evaluated histopathology. All authors read and approvaed the final manuscript.
